# The Interaction between Regulatory T Cells and NKT Cells in the Liver: A CD1d Bridge Links Innate and Adaptive Immunity

**DOI:** 10.1371/journal.pone.0027038

**Published:** 2011-11-02

**Authors:** Jing Hua, Shuwen Liang, Xiong Ma, Tonya J. Webb, James P. Potter, Zhiping Li

**Affiliations:** 1 Shanghai Renji Hospital, Jiaotong University School of Medicine, Shanghai Institute of Digestive Disease, Shanghai, China; 2 Department of Medicine, Johns Hopkins University, Baltimore, Maryland, United States of America; 3 Department of Microbiology & Immunology, University of Maryland School of Medicine, Baltimore, Maryland, United States of America; Ulm University, Germany

## Abstract

**Background/Aims:**

Regulatory T cells (Tregs) and natural killer T (NKT) cells are two distinct lymphocyte subsets that independently regulate hepatic adaptive and innate immunity, respectively. In the current study, we examine the interaction between Tregs and NKT cells to understand the mechanisms of cross immune regulation by these cells.

**Methods:**

The frequency and function of Tregs were evaluated in wild type and NKT cell deficient (CD1dko) mice. *In vitro* lymphocyte proliferation and apoptosis assays were performed with NKT cells co-cultured with Tregs. The ability of Tregs to inhibit NKT cells *in vivo* was examined by adoptive transfer of Tregs in a model of NKT cell mediated hepatitis.

**Results:**

CD1dko mice have a significant reduction in hepatic Tregs. Although, the Tregs from CD1dko mice remain functional and can suppress conventional T cells, their ability to suppress activation induced NKT cell proliferation and to promote NKT cell apoptosis is greatly diminished. These effects are CD1d dependent and require cell to cell contact. Adoptive transfer of Tregs inhibits NKT cell-mediated liver injury.

**Conclusions:**

NKT cells promote Tregs, and Tregs inhibit NKT cells in a CD1d dependent manner requiring cell to cell contact. These cross-talk immune regulations provide a linkage between innate and adaptive immunity.

## Introduction

The liver is constantly exposed to food antigens, endotoxins, and pathogens that are brought from the intestines via the portal blood supply. As a result, the liver must generate vigorous immune responses. This is accomplished by a large number of hepatic Kupffer cells, natural killer (NK) cells and natural killer T (NKT) cells, which are components of innate immunity. Meanwhile, the liver maintains immune tolerance to avoid inappropriate immune activation, e.g. orthotopic liver transplantation results in the long-term allograft survival without immunosuppression [1]. One important component of hepatic immune tolerance is represented by lymphocytes with regulatory functions, including regulatory T cells (Tregs) and NKT cells [2], [3].

Tregs are a group of heterogenous T cells that belong to the adaptive immune system and are actively engaged in the negative control of a variety of immune responses, including transplant tolerance [4], viral hepatitis [5], autoimmune hepatitis [6], and hepatocellular carcinoma [7]. Tregs are usually CD4^+^CD25^+^ and express forkhead box protein 3 (Foxp3), a specific marker and the master transcriptional regulator of Treg development and function [8]. In the liver, the over-regulation/suppression of Tregs has been shown to contribute to chronic HBV/HCV infections [5], [9] and hepatocellular carcinoma [7]. In contrast, inadequate Treg regulation contributes to autoimmune hepatitis [6], primary biliary cirrhosis [10], and acute rejection of transplant grafts [11].

NKT cells are components of the innate immune system. They recognize glycolipid antigens bound to the MHC class I-like molecule, CD1d, noncovalently associated with β2 micro globulin on various antigen presenting cells [12]. NKT cells respond to antigen presentation by secreting large amounts of cytokines, which in turn stimulate the proliferation and differentiation of a variety of other immune cells that participate in the innate or adaptive response [13]. In the liver, more than 95% of the NKT cells are invariant NKT cells that predominantly express a conserved αβ T cell receptor [14], [15]. These cells originate in the thymus, but predominately accumulate in the liver, where they are thought to be responsible for the regulatory function by secretion of both pro-inflammatory and anti-inflammatory cytokines [16], [17], [18].

Previously, it has been shown that there is evidence of cross-talk between Tregs and NKT cells [19]. NKT cells secrete IL-2 and IL-4 that induce Tregs proliferation [20], [21]. In addition, NKT cells also regulate the homing of Tregs to the liver [22]. Conversely, Tregs can also inhibit NKT cell proliferation and cytokine production *in vitro* [23]. However, there is little evidence of their interaction in the liver. There is also little knowledge of the mechanism by which Tregs exert their regulatory role on NKT cells. Thus, in the current study, we have examined the role of Tregs in hepatic NKT cell regulation, including the mechanism of regulation and *in vivo* implications of such regulation.

## Methods

### Ethics statement

All animal experiments fulfilled NIH and JHU criteria for the humane treatment of laboratory animals and were approved by the Johns Hopkins Animal Care and Use Committee (MO07M195).

### Animal experiments

Adult (age 6–8 week) male wild type (wt) C57BL6 mice were purchased from Jackson Laboratories (Bar Harbor, ME). CD1d knock out (CD1dko) mice were originated in Dr. Albert Bendelac’s lab and back crossed to C57BL/6 background more than 10 generation. All mice were maintained in a temperature- and light-controlled facility, and permitted *ad libitum* consumption of water and pellet chow. To evaluate the role of IL-2 on Treg regulation, some wt and CD1dko also received recombinant mouse IL-2 (2×10^5^ IU, i.p., BD Pharmingen) on days 1, 3, 5, 7, as previous described [24], and were evaluated on day 8 for their hepatic and splenic Treg contents. To activate NKT cell *in vivo*, wt mice were injected either vehicle or 2 µg of α-galactosylceramide (α-Galcer, ALEXIS Biochemical, San Diego, CA) via tail vein 3 days before harvesting the liver and collecting the blood.

### Isolation and labeling of hepatic mononuclear cells (HMNCs) and splenocytes

Mouse livers were perfused briefly with sterile saline solution to remove blood cells, then carefully removed and minced. The liver and spleen was homogenized and passed through a 70-micron nylon mesh to remove connective tissue. HMNCs were then isolated with Percoll gradient as previously described [25]. Splenocytes were isolated after removal of red blood cells. Cells were then labeled with a CD1d tetramer (NIH tetramer facility) loaded with a ligand (PBS-57, an analogue of α-GalCer) or anti-mouse fluorescent antibodies against CD3, CD25, CD4, CD62L, CD103 (Pharmingen, San Diego, CA). For apoptosis assays, cells were stained with Annexin V and the vital dye 7-aminoactinomycin D (7-AAD). For intracellular staining, cells were labeled with surface antibody, as described above, permeabilized and then stained with antibodies against Foxp3 (eBioscience, San Diego, CA), IL-2 or CTLA-4 (Pharmingen) according to the manufacturer's instructions. After labeling, cells were evaluated by flow cytometry (Becton Dickinson, Palo Alto, CA), and the data were analyzed using Cell Quest software (Becton Dickinson). For intracellular IL-2 staining only, cells were also pre-incubated with a leukocyte activation cocktail first, which includes phorbol 1,2-myristate 1,3-acetate (PMA, 50 ng/ml), ionomycin (500 ng/ml), and GolgiPlug (1 µl/ml) for 5 hrs.

### Cell purification and adoptive transfer

NKT cells were isolated from livers of wt mice. After isolation, HMNCs were labeled with surface marker antibodies (anti-TCRβ and anti-NK1.1). NKT cells (NK1.1^+^, TCRβ^+^) were isolated through FACSVantage SE high speed sorter (Becton Dickenson). The majority of those NKT cells (NK1.1^+^, TCRβ^+^) were CD1d tetramer positive, as shown in our previous study [17]. After isolation, NKT cells were cultured with RPMI media supplemented with 2 mM L-glutamine. 100 U/ml penicillin, 100 µg/ml streptomycin and 10% FCS. CD4^+^CD25^+^ Tregs and CD4^+^CD25^-^ effector T cells (Teffs) were isolated from the spleen of wt or CD1dko mice by using a MACS regulatory T cell isolation kit (Miltenyi Biotec, Auburn, CA). The purity of the cell separation was ∼95%, as assessed by flow cytometry.

After isolation, 1.5×10^6^ Tregs or Teffs were adoptively transferred to each recipient wt mouse via tail vein injection. 24 hour after adoptive transfer, the recipient mice were injected with 2 µg of α-Galcer or vehicle as described above to activate NKT cells. Liver tissue and blood were collected 3 days later.

### In vitro Treg suppression assays

Two types of Treg suppression assay were performed. In one assay, purified wt and CD1dko Tregs were co-cultured with Teffs (5×10^4^/well) that were stimulated with anti-CD3 (1 µg/ml) in the presence of APCs (mitomycin C (50 µg/ml) treated splenocytes, 5×10^4^/well). In the other assay, purified wt and CD1dko Tregs were co-cultured with purified NKT cells (5×10^4^/well) that were stimulated with mitomycin C treated splenocytes (5×10^4^/well) loaded with α-GalCer (100 ng/ml for 3 hrs. In both assays, APCs (either treated with mitomycin-C alone for Teffs, or treated with α-GalCer and mitomycin-C for NKT cells) were washed extensively before co-cultured with either NKT cells or Teffs in the presence of untreated Tregs in round-bottom 96-well plates with RPMI medium. Cells were co-cultured for 3 days, at the last 18 h, 0.5 Ci [^3^H] thymidine was added to the culture. Cell proliferation was determined by incorporation of [^3^H] thymidine. Culture media were collected for cytokine determination. In separate studies, Tregs (2×10^5^/well) with APCs (2×10^5^/well) were placed in transwell (Millipore, Billerica, MA) chambers and cultured with NKT (2×10^5^/well) cells in the present of APCs (2×10^5^/well) as described above.

### Tregs and NKT cells conjugation assay

Vα14^+^ mouse CD1d-specific NKT hybridoma (DN32.D3) were labeled with CD1d tetramer, then incubated with freshly isolated Tregs (1∶1) from WT mice or CD1d ko mice that were labeled with anti-CD25 mAb. After washing and fixation, cells were analyzed by FACS. CD1d-tetramer^+^CD25^+^ cells indicated the conjugation between NKT and Tregs. For CD1d blocking assays, anti-CD1d mAb (10 µg/ml) were added to culture. In separate experiments, purified NKT cells and Tregs were labeled with 20 µM CellTracker green dye and 5 µM CellTracker red dye, respectively (Invitrogen, Eugene, OR), and were co-cultured for 24 hours in an incubator chamber connected to a confocal microscope. The images were recorded every 30 min and analyzed using velocity super-64 software (Improvision, PerkinElmer). Pearson's correlation coefficients described as the correlation of intensity distribution between red and green channels were used to assess NKT cell and Treg conjugation [26].

### RNA Isolation and Evaluation of Hepatic Gene Expression

RNA was isolated as described previously [27], [28]. cDNA was synthesized from 5 µg of total RNA using oligo(dT) as a template and the SuperScriptII kit (Invitrogen). Quantified PCR amplifications were performed using TagMan Gene Expression Assays PCR Master Mix and primer sets (Applied Biosystems, Foster City, CA). Negative controls were performed without cDNA in the reaction mixture. The results were normalized against glyceraldehyde-3-phosphate dehydrogenase gene expression.

### Liver Histology, serum alanine aminotransferase (ALT) and ELISA

Thin slices of liver tissue were stained with hematoxylin. Serum ALT was determined by the spectrophotometric method as previously described [29]. IL-4, IFN-γ and IL-2 levels in culture media were measured with standard ELISA kits according to the manufacture's instruction (eBioscience).

### Statistical analysis

All values are expressed as mean ± SD. Treatment related differences were evaluated by ANOVA. The paired-individual means were compared by t-test. *P* values of less than 0.05 were considered statistically significant.

## Results

### Reduction of hepatic Tregs in NKT deficient mice

**Figure 1:**
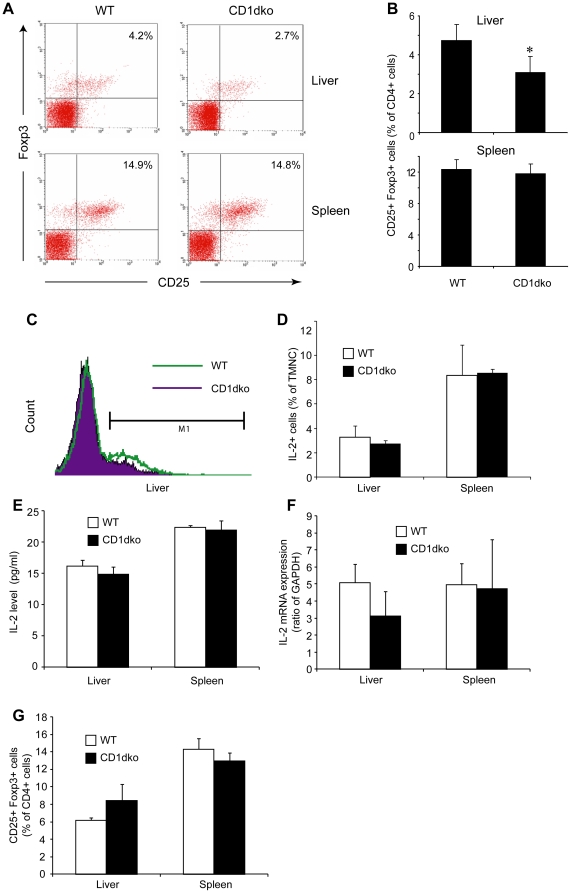
Reduction of hepatic Tregs in CD1d deficient mice. Hepatic mononuclear cells (HMNC) and splenic cells were isolated from wt and CD1dko C57BL6 mice. Tregs were identified by CD4, CD25, and Foxp3 staining. (n = 7 each group). **A**) Representative dot plots of Tregs (CD25^+^ Foxp3^+^ cells, gated CD4^+^ cells) from liver (upper panels) and spleen (lower panels). **B**) Mean (±SD) percentage of splenic and hepatic Tregs among CD4 cells from wt and CD1dko mice. **C**) Representative histogram of intracellular IL-2 staining of HMNC from wt (open bar) and CD1dko (solid bar) mice. **D**) Mean (±SD) value of intracellular IL-2 expression of total mononuclear cells (TMNC) in the liver and the spleen of wt and CD1dko mice. **E**) Mean (±SD) level of IL-2 released from HMNC and splenic cells of wt and CD1dko mice measured by ELISA. **F**) Mean (±SD) percentage of IL-2 mRNA expression in the liver and the spleen of wt and CD1dko mice determined by quantitative PCR. **G**) Mean (±SD) percentage of splenic and hepatic Tregs in wt and CD1dko mice after they received recombinant IL-2. *p<0.05 vs wt mice.

There are several reports of cross talk between NKT cells and Tregs [30], [31], [32]. However, there is little evidence of whether NKT cell deficiency leads to a reduction in Treg number or function. In this study, we first examined the basal number of Tregs in NKT cell deficient (CD1dko) mice. There was a significant decrease of Tregs in the livers of CD1dko mice (Fig. 1 A and B). However, the reduction in Tregs was limited to the liver. The number of Tregs in the spleen of CD1dko mice was similar to those of wt mice (Fig. 1A and B). Because liver has a high percentage of IL-2 producing NKT cells, which is necessary for Treg development [20], we next evaluated hepatic IL-2 expression in CD1dko and wt mice. Intracellular cytokine staining showed a slightly decrease in IL-2 expression among HMNC from CD1dko mice, while IL-2 expression in the spleens of wt and CD1dko mice was similar (Fig. 1C and D). There was a trend of reduction in IL-2 transcription and translation level, as shown by qPCR and ELISA (Fig. 1E and F), although they didn't reach statistical significance. Hepatic Treg number returned to normal levels after CD1dko mice received exogenous IL-2 (Fig. 1G). Taken together, it is likely that local IL-2 secretion controlled by NKT cells regulates the accumulation of Tregs in the liver and leads to decreased hepatic Tregs in CD1dko mice.

### Tregs display CD1d dependent NKT cell suppression

**Figure 2:**
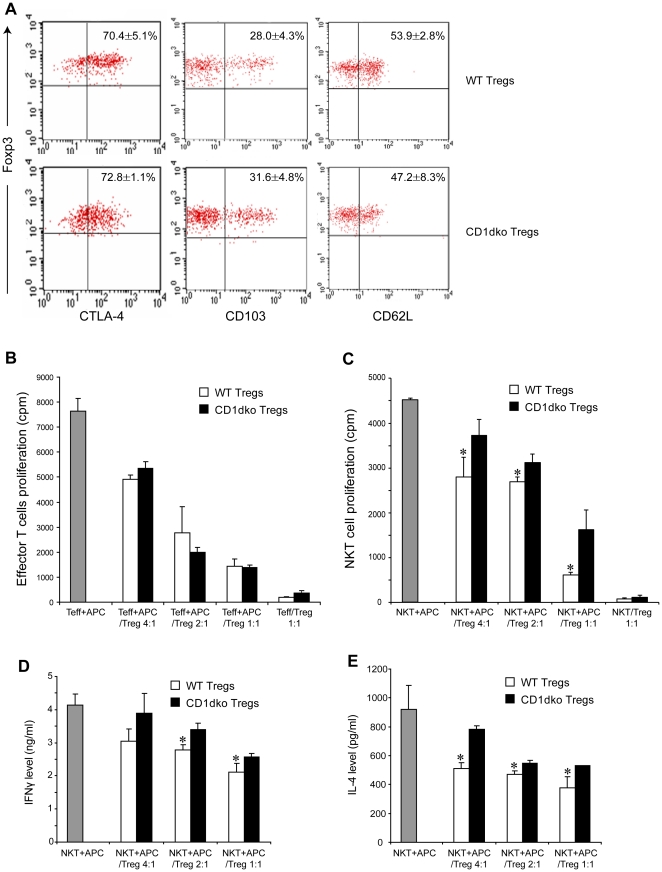
CD1d deficient Tregs have a reduced capacity to suppress NKT cells. Tregs (CD4^+^ CD25^+^) were isolated from the spleen of wt and CD1dko mice. NKT cells (NK1.1^+^, TCRβ^+^) and Teffs (CD4^+^ CD25^-^) were isolated from the liver and the spleen of wt mice, respectively. **A**) Representative dot plots of isolated Tregs from each animal of one experiment. Mean (±SD) results were listed on the graph. Tregs from wt and CD1dko mice share similar Treg surface markers. **B**) Tregs suppress Teff proliferation assay. Teffs (5×10^4^/well) were co-cultured with different amount of either wt or CD1dko Tregs as labeled on the graph in present of APC (mitomycin C-treated wt splenocytes, 5×10^4^/well) and anti-CD3. Mean (±SD) results were graphed. **C**) Tregs suppress NKT cell proliferation assay. NKT cells (5×10^4^/well) were co-cultured with different amount of either wt or CD1dko Tregs as labeled on the graph in present of APC (α-Galcer-loaded and mitomycin C-treated wt splenocytes, 5×10^4^/well). The APCs were washed extensively to remove excessive α-Galcer before added to the co-culture. Mean (±SD) results were graphed. *p<0.05 vs wt mice. **E, F**) Tregs suppress NKT cell function assays. NKT cells were isolated and co-cultured with APCs and Tregs from wt and CD1dko mice, as described above. IFN-γ (**E**) and IL-4 (**F**) released to the culture media were determined by ELISA. Mean (±SD) results were graphed. (**B**–**F**, n = 3/exp, and the experiments were repeated three times). *p<0.05 vs wt mice.

A previous study has shown that human Tregs suppress NKT cell proliferation and cytokine production [23]. To better understand the mechanism by which Tregs regulate NKT cell function, we performed lymphocyte suppression assays using freshly isolated Tregs. CD4^+^ CD25^+^ Tregs were sorted from the spleens of either wt or CD1dko mice. Given that Foxp3 represents a single definitive Treg marker [8], we determined its expression on isolated CD4^+^ CD25^+^ Tregs and found that the majority of these cells in both groups were Foxp3^+^ cells (Fig. S1A). Other functional markers such as CTLA-4, CD62L and CD103 were also similarly expressed on Tregs from either wt or CD1dko mice (Fig 2A). Notably, Tregs from wt mice express CD1d, which was absent on Tregs from CD1dko mice (Fig. S1B). Nevertheless both groups displayed a similar ability to suppress conventional T cell proliferation (Fig 2B). Next, we evaluated whether Tregs can also suppress NKT proliferation. Purified wt NKT cells were cultured with α-Galcer-loaded splenocytes in the presence or absence of Tregs from either wt or CD1dko mice. Tregs from wt mice demonstrated much stronger inhibitory capacity on NKT cell proliferation than those from CD1dko mice (Fig 2C). Because wt Tregs express CD1d (Fig. S1B), to rule out the possibility that NKT cells maybe stimulated by wt Tregs in association of either endogenous or exogenous ligands (α-Galcer) presented by CD1d molecules, NKT cells were cultured with wt Tregs in the absence of splenocytes. No NKT cell proliferation was observed (data not shown). In addition, the α-Galcer-pulsed splenocytes were washed extensively to remove the residual α-Galcer before being added to the culture system. These results indicate that Tregs alone cannot effectively stimulate NKT cells. Furthermore, when NKT cells were activated by anti-CD3 mAbs, which do not require CD1d-mediated ligand presentation, a similar inhibitory effect was observed on NKT cells proliferation by wt Tregs but much less by CD1dko Tregs (Fig. S2). Taken together, these results demonstrate that Tregs inhibit activation-induced NKT cell proliferation in a CD1d-dependent manner.

Upon stimulation NKT cells rapidly produce many cytokines, including IFN-γ and IL-4. Next, we evaluated whether Tregs could also suppress activation-induced cytokine production by NKT cells. NKT cells were co-incubated with α-Galcer loaded APC in the presence of Tregs isolated from either wt or CD1dko mice. Co-culture supernatants were harvested and cytokine production was measured by standard cytokine ELISA. We found that, in addition to their ability to inhibit NKT cell proliferation, CD1d expressing Tregs also are much more effective at suppressing NKT cell function, compare to CD1d deficient Tregs (Fig. 2D, E).

### The suppression of Tregs by NKT cells requires CD1d mediated cell to cell contact

**Figure 3:**
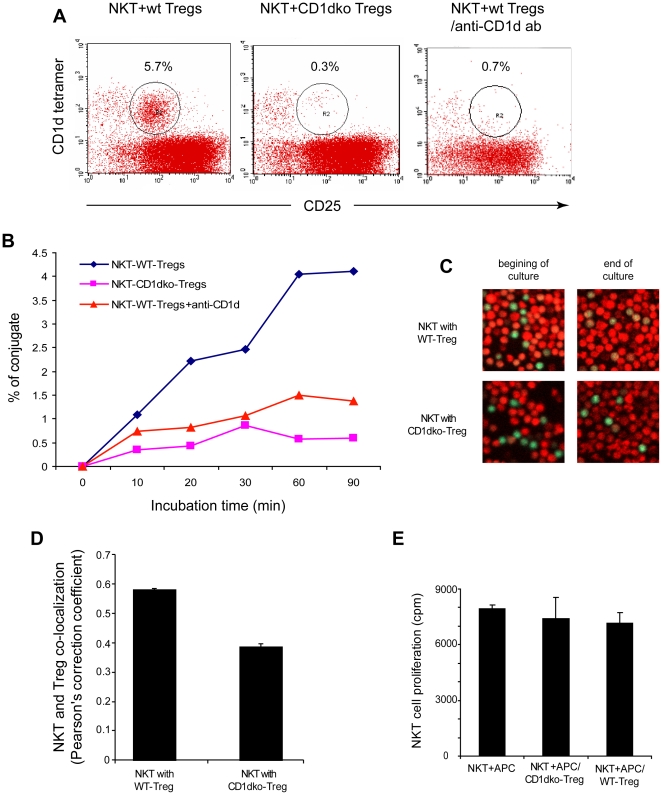
CD1d dependent conjugation of Tregs and NKT cells. Tregs were isolated and co-cultured with either NKT cell hybridomas (**A** and **B**) or purified primary NKT cells (**C-E**). **A**) Representative dot plots of cells after co-culture for 60 minutes. CD25 represents Treg marker and CD1d tetramer represents NKT cell marker. CD25^+^ CD1d tetramer^+^ cells indicated conjugation of Tregs and NKT cells. **B**) Time course of Treg and NKT cell conjugation after co-culture. **C, D**) NKT cells and Tregs were labeled with green and red dye, respectively, and co-cultured while their co-localization was measured with confocal microscopy. **C)** Representative cofocal images showed at the beginning and the end of culture. NKT cells only conjugated with wt Tregs as indicated as yellow colored double labeling. **D)** Pearson's correlation coefficients were used to determine the conjugation. The coefficient of >0.5 indicated conjugation and <0.5 indicated no conjugation. **E**) NKT cell proliferation assays similar to those described in Fig. 2C except the Tregs were placed in transwells. NKT:Treg = 1∶1, (**B** – **E**, n = 3/exp, and the experiments were repeated three times).

CD1d molecules present ligands to NKT cells via conjugation with an invariant T cell receptor. Our results indicated that the suppressive function of Tregs on NKT cells was CD1d-dependent. We co-incubated Vα14^+^ NKT hybridoma with Tregs from wt mice and CD1dko mice to examine whether Tregs suppression of NKT cells requires conjugation and cell to cell contact. After co-culturing NKT hybridoma and Tregs, there was dual-expression of both Treg and NKT markers, indicating their conjugation (Fig. 3A, B). Importantly, we found that only Tregs from wt mice, not CD1dko mice, conjugated with NKT hybridoma. Furthermore, conjugate formation was blocked by the addition of anti-CD1d mAb (Fig. 3A, B). To further confirm the conjugation between NKT cells and Tregs, we labeled purified primary NKT cells and Tregs with green and red dye, respectively, and co-cultured them while measuring their co-localization with confocal microscopy (Fig. 3C). Using Pearson's correlation coefficients as described previously [26], we found that NKT cells conjugated with wt Tregs (>0.5), but not with CD1d deficient Tregs (<0.5) (Fig. 3D). In addition, we examined whether Treg mediated suppression of NKT cell function was contact dependent. We found that when Tregs were placed in transwells and cultured above primary NKT cells, their ability to suppress NKT cells was abolished (Fig. 3E). These data provide some indication that the suppression by Tregs of NKT cells is both CD1d and cell-contact dependent.

### Tregs induce NKT cell apoptosis

**Figure 4:**
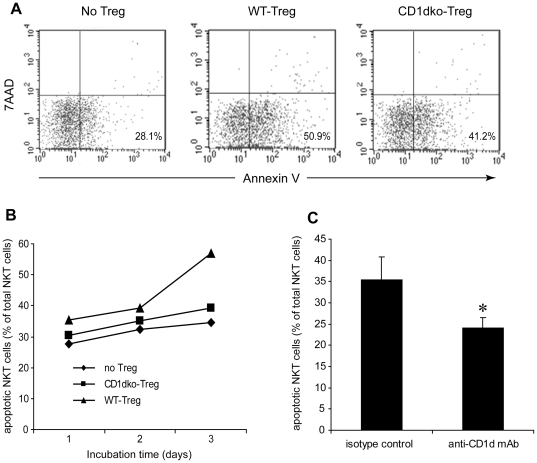
CD1d-mediated Treg-induced NKT cell apoptosis. NKT cells and Tregs were isolated and co-cultured as described in Figure legend 2. NKT cell apoptosis was measured by AnnexinV staining with concurrent incubation of 7-AAD to assess cell necrosis. **A**) Representative dot plot of NKT cell (gated on CD1d-tetramer+ cells) apoptosis assay after co-cultured for 3 days. Apoptotic NKT cells are defined as Annexin-V^+^/7-AAD^−^. **B**) Time-course analysis of NKT cell apoptosis induced by Tregs isolated from wt and CD1dko mice. (n = 3 each group). **C**) Blocking of CD1d engagement completely abrogated Treg-mediated NKT cell apoptosis. Purified Tregs from wt mice were first incubated with anti-CD1d mAb or isotype control (10 g/ml, 1B1 clone, BD Biosciences) for 2 hours. After extensive washing, the Tregs were then co-cultured with NKT cells for 72 hours as described above. Mean (±SD) of three experiments were graphed. (n = 3/exp, and the experiments were repeated 3 times) *p<0.05 vs isotype control.

It has previously been described that Treg based suppression involved both inhibition of target cell proliferation and induction of apoptosis of target cells [33]. Thus, we evaluated whether Tregs also induce apoptosis in activated NKT cells. Primary NKT cells were activated with α-Galcer loaded APC, and then co-cultured with Tregs from either wt or CD1dko mice, as previously described. We then used Annexin-V staining to evaluate the level of NKT cell apoptosis by simultaneously incubating the cells with 7-AAD to assess cellular necrosis. Apoptotic NKT cells were defined as Annexin-V^+^/7-AAD^−^. There was a significant increase in the apoptosis of NKT cells induced by wt Tregs, whereas NKT cells apoptosis was less frequent with Tregs from CD1dko mice (Fig. 4A). Treg induced NKT cell apoptosis increased with time (Fig. 4B) and could be blocked by anti-CD1d mAb (Fig. 4C). These results further illustrated that Tregs may exert their regulatory function on NKT cells via a CD1d dependent mechanism.

### Tregs inhibit NKT cell mediated hepatitis

**Figure 5:**
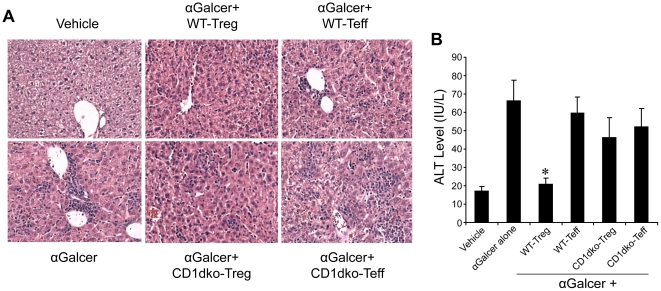
Tregs inhibit NKT cell mediated hepatitis. Tregs (CD4^+^ CD25^+^) and Teff (CD4^+^CD25^−^) were isolated from the spleen of either wt or CD1dko C57BL6 mice and adoptively transferred (1.5×10^6^ Tregs for each recipient mouse) into wild-type C57BL6 mice. 24 hour after adoptive transfer, the recipient mice were injected with α-Galcer to induce hepatits. Liver tissue and blood were collected 3 days later. **A**) Representative H&E stain of liver histology. **B**) Mean (±SD) serum ALT levels of four experiments, *p<0.01 vs other groups treated with α-Galcer.

NKT cells are known to mediate several forms of hepatitis [34], [35]. To evaluate whether the Tregs-induced suppression of NKT cells has any biological relevance, wt mice were injected with α-Galcer to elicit hepatitis, then splenic Tregs from wt or CD1dko mice were adoptively transferred into these mice. Our previous study shows homing of transferred Tregs in the liver using the same method [36]. Wt Tregs, but not CD1dko Tregs, nor Teff, significantly reduced hepatic inflammation induced by α-Galcer as reflected by liver histology (Fig. 5A) and serum ALT levels (Fig. 5B). These studies indicate that Tregs play an important biological role in the regulation of NKT cell activation and NKT cell mediated inflammation. In addition, these data demonstrate that Treg mediated suppression of NKT cells is CD1d dependent *in vivo*.

## Discussion

Tregs and NKT cells are two distinct lymphocyte subsets. NKT cells are enriched in the liver where they are the major component of the innate immune defense. They recognize glycolipid antigens presented by CD1d and respond to infections or inflammation prior to the conventional adaptive immune responses. Hepatic NKT cells include groups of functionally distinct subsets that can both promote and suppress immune responses, and have been implicated in the pathogenesis of a wide variety of autoimmune and inflammatory diseases [37], [38]. The regulatory function of NKT cells is thought to be due to their ability to produce large amounts of cytokines, particularly IL-4, IL-10 and IL-13 [39]. Here we show that NKT cells are likely to also promote the Treg population in the liver, possible through IL-2, because NKT cell deficient CD1dko mice have reduced IL-2 level and less Tregs in the liver (Fig. 1). This provides another mechanism by which NKT cells can regulate the immune response.

As a part of the adaptive immune response, Tregs recognize antigens bound to MHC class-II molecules and mediate tolerance through contact-dependent suppression of effector-cell proliferation [40]. Dysfunction of Tregs has been linked to autoimmune hepatititis [41]. Cross-talk between NKT cells and Tregs has also been described [19]. In fact, hepatic NKT cell number appears to be increased in association with decreased hepatic Treg number in some autoimmune liver diseases [10], [42]. These observations suggest that the dysregulation of NKT cell and Treg interactions may contribute to the pathogenesis of autoimmune liver disease. However, the mechanism of NKT cell and Treg cross regulation has not been well characterized.

In this manuscript, we use both an *in vivo* animal model and an *in vitro* cell culture system to study immune regulation and CD1d-dependent cross-talk between NKT cells and Tregs in the liver. We show that NKT cells induce or recruit Tregs to the liver, because NKT cell deficiency (CD1dko mice) results in a reduction in the population of hepatic Tregs. The decrease in Tregs in the liver of NKT cell deficient mice are likely due to decreased hepatic IL-2 and IL-4, major cytokines secreted by NKT cells. We also demonstrated that Tregs express high levels of CD1d molecules on their surface. Furthermore, we found that Tregs effectively inhibited NKT cell proliferation through the induction of apoptosis. This inhibitory effect was CD1d dependent, since CD1dko Tregs have normal inhibitory activities on classical effector T cells, while their inhibitory activities on NKT cells were diminished and this effect could be blocked with CD1d-specific mAbs. The inhibitory effect was also cell-cell contact dependent. These observations provide insights to the pathogenesis of many autoimmune related liver diseases. From a clinical point of view, many infection or inflammation can result in hepatic NKT cell activation and proliferation as part of the innate immune response. Once activated, NKT cells secrete IL-2 and IL-4 to recruit or induce Tregs to the liver as part of the adaptive immune response, which, in turn, inhibits NKT cell proliferation and promote NKT cell apoptosis to limit the NKT cell mediated inflammatory response. This feedback mechanism prevents excessive inflammation. Any dysregulation of NKT cell and Treg cross-talk may contribute to the pathogenesis of immune mediated liver diseases. Our animal model, with NKT cell-mediated hepatitis, indicates the importance of this cross-talk and regulation. However, the molecular mechanism of Treg and NKT cell interaction is still unclear. The receptor complex and intracellular signaling pathway that mediate Treg and NKT cell interaction are still under investigation.

It should be noted that, although the suppressive ability of wt Tregs was much higher than that of CD1dko Tregs, the latter still exerted some inhibitory effects on NKT cells (Fig 3). Multiple mechanisms behind Tregs suppressive function have been proposed, including contact-dependent suppression of effector-cell proliferation involving IL-10 and transforming growth factor (TGF)– β [40], and modulation of dendritic cell maturation and function [43]. Therefore, it is possible that other suppressive mechanisms in addition to the CD1d mediated-cell to cell contact also play role in the cross-talk between Tregs and NKT cells. These other mechanisms are currently under investigation.

In summary, we show that NKT cells and Tregs, two major regulatory sub-populations of lymphocytes that belong to the innate and adaptive immune system, interact with each other in the liver to generate an effective, but tightly-regulated immune response. This provides a novel concept of hepatic immune regulation and the possible pathogenesis of immune mediated liver diseases.

## Supporting Information

Figure S1 **A)** Representative histogram of Foxp3 intracellular staining that showed the majority of isolated CD4^+^ CD25^+^ cells are Tregs with similar distribution between wt and CD1dko mice. **B)** Representative histogram of CD1d staining on Tregs from wt and CD1dko mice. Tregs from wt mice express CD1d, which was absent on Tregs from CD1dko mice.
(TIF)

Figure S2 NKT cells were isolated from the liver of wt mice. Tregs (5×10^4^/well) were purified from the spleen of either wt or CD1dko mice, and co-cultured with NKT cells (5×10^4^/well) in the present of anti-CD3 mAb and mitomycin C treated splenocytes (5×10^4^/well). NKT cell proliferation was determined by incorporation of [^3^H] thymidine. Mean (±SD) results of triplicates experiments were graphed. ^a^p<0.01 vs no-Treg group, ^b^p<0.01 vs CD1dko-Treg group.
(TIF)
