# Supplementary figures and images for: The Interaction between Regulatory T Cells and NKT Cells in the Liver: A CD1d Bridge Links Innate and Adaptive Immunity
Source: PLoS One. 2011 Nov 2;6(11):e27038. doi: 10.1371/journal.pone.0027038 (PMC3206882; doi:10.1371/journal.pone.0027038)

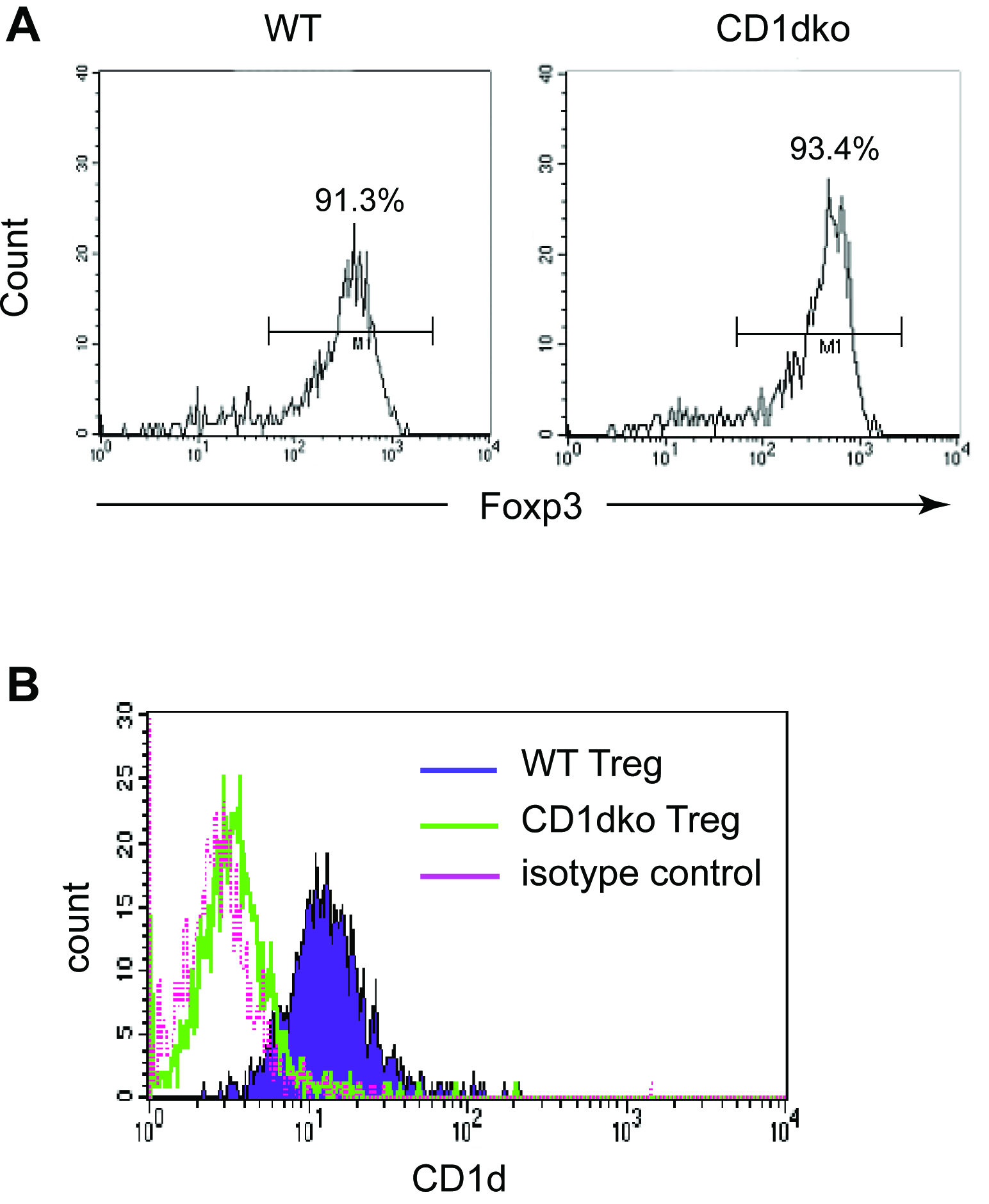

Supplement: Figure S1 — A) Representative histogram of Foxp3 intracellular staining that showed the majority of isolated CD4+ CD25+ cells are Tregs with similar distribution between wt and CD1dko mice. B) Representative histogram of CD1d staining on Tregs from wt and CD1dko mice. Tregs from wt mice express CD1d, which was absent on Tregs from CD1dko mice. (TIF) [file pone.0027038.s001.tif]

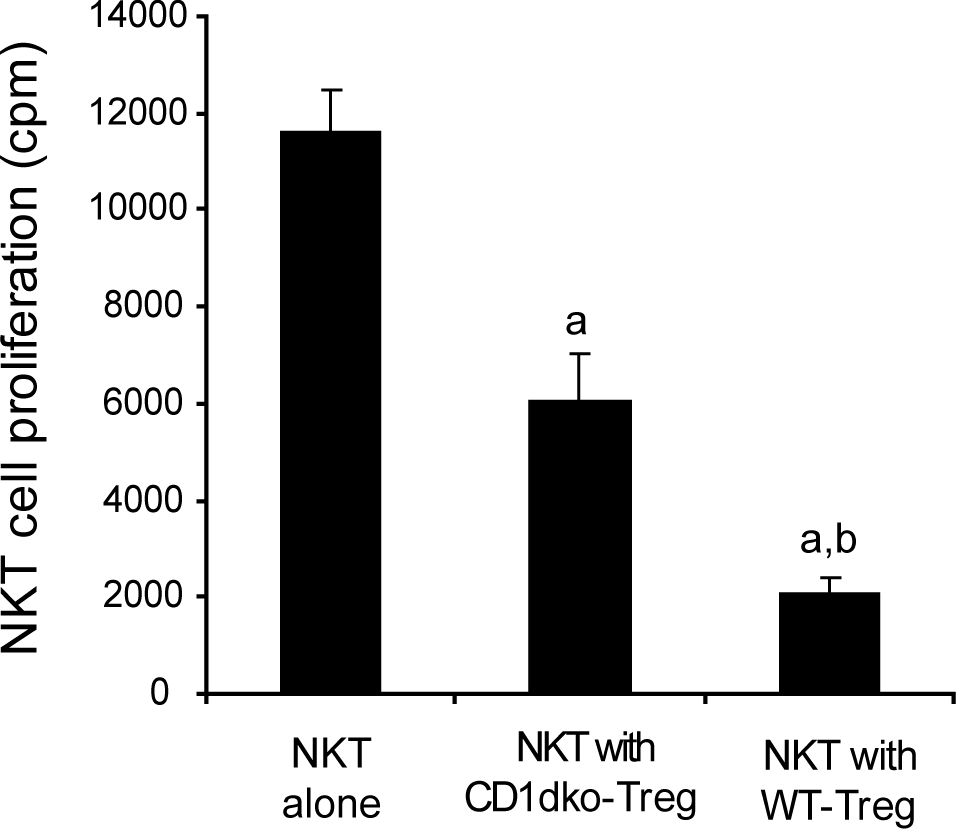

Supplement: Figure S2 — NKT cells were isolated from the liver of wt mice. Tregs (5×10^4^/well) were purified from the spleen of either wt or CD1dko mice, and co-cultured with NKT cells (5×10^4^/well) in the present of anti-CD3 mAb and mitomycin C treated splenocytes (5×10^4^/well). NKT cell proliferation was determined by incorporation of [3H] thymidine. Mean (±SD) results of triplicates experiments were graphed. ap<0.01 vs no-Treg group, bp<0.01 vs CD1dko-Treg group. (TIF) [file pone.0027038.s002.tif]
